# Expression of *MDR1*, *HIF-1α *and *MRP1 *in sacral chordoma and chordoma cell line CM-319

**DOI:** 10.1186/1756-9966-29-158

**Published:** 2010-12-08

**Authors:** Zhengang Ji, Hua Long, Yunsheng Hu, Xiuchun Qiu, Xiang Chen, Zhao Li, Degang Fan, Baoan Ma, Qingyu Fan

**Affiliations:** 1 Department of Orthopedic Surgery, Orthopedics Oncology Institute of Chinese PLA, Tangdu Hospital, Fourth Military Medical University, Xi'an, Shaanxi Province, 710038, P. R. China

## Abstract

**Background:**

Chordoma was a typically slow-growing tumor. The therapeutic approach to chordoma had traditionally relied mainly on surgical therapy. And the main reason for therapeutic failure was resistance to chemotherapy and radiotherapy. However the refractory mechanism was not clear. The aim of this study was to investigate the expression of three genes (*MDR1*, *HIF-1α* and *MRP1*) associated with resistance to chemotherapy and radiotherapy in chordoma and chordoma cell line CM-319.

**Materials and methods:**

Using immunohistochemical techniques, the expression of MDR1, HIF-1α and MRP1 was investigated in 50 chordoma specimen. Using RT-PCR and Western blot, the expression of MDR1, HIF-1α and MRP1 was investigated in chordoma and chordoma cell line CM-319.

**Results:**

Expression of MDR1, HIF-1α and MRP1 was observed in 10%, 80% and 74% of all cases, respectively. Expression of MRP1 was correlated with HIF-1α. On the other hand, expression of MDR1 was not correlated with the expression of HIF-1α or MRP1. The expression of HIF-1α and MRP1 was observed, but MDR1 was not observed in chordoma and CM-319.

**Conclusion:**

Expression of HIF-1α and MRP1 was observed in most chordoma specimen and CM-319 cell line; expression of HIF-1α correlated with MRP1. HIF-1α and MRP1 may play a role in the multidrug resistance of chordoma to chemotherapy.

## Introduction

Chordoma, a primary malignant tumor of the skeleton, was considered to develop from a remnant of notochordal cells in the midline skeletal axis [1]. The most common sites are the skull base and the sacrococcygeal region. It is typically slow-growing tumor, and initial symptoms are usually related to local progression of the disease with subsequent compression of adjacent structures. The natural course of chordoma is quite grim; most patients do not survive 10 years because of high local recurrence rates [2,3]. The therapeutic approach to chordoma has traditionally relied heavily on surgical control. More recently, radiation therapy has been demonstrated to be a valuable modality for local control, particularly with the advent of charged particle radiotherapy. Medical therapy continues to be suboptimal in chordoma which is relatively refractory to cytotoxic chemotherapy. The main reason for therapeutic failure in cases of chordoma involves resistance to chemotherapy and radiotherapy. The refractory reason to chemotherapy and radiotherapy may be associated to the over-expression of some multidrug resistance related genes and hypoxia inducible factor-1α. These factors could also provide potential targets for studies on novel therapeutic procedures.

Multidrug resistance is a frequent cause of treatment failure in cancer patients. One mechanism of MDR is over-expression of ATP-binding cassette (ABC) transporter proteins that function as a drug efflux pump. These ABC transporter proteins include P-glycoprotein (P-gp) [4], which is a member of the multidrug resistance-associated protein (MRP) family, the recently identified breast cancer resistance protein (BCRP), and the lung resistance-related vault protein (LRP) identified as the major vault protein (MVP) which are also associated with MDR.

The hypoxia-inducible factor (*HIF*) is an alpha (α)/beta (β) heterodimeric DNA binding complex and directs extensive transcriptional responses involving the induction of genes relevant to tumor progression, such as angiogenesis, metabolism, cellular growth, metastasis, and apoptosis. *HIF-1α *has emerged as an attractive target for cancer therapy [5,6].

Over-expression of P-gp and MRP is generally believed to be the mechanism responsible for MDR of tumor cells. Hypoxia is a common feature of many malignant tumors. HIF-1 is a key factor in altering the biological characteristics of tumors [7-9]. Many studies indicate that hypoxia helps to improve chemotherapy and radiotherapy resistance of tumors [10-12].

To our knowledge, the mechanism of multidrug resistance to chemotherapy remained largely unknown in chordoma. The present study aimed to investigate the relationship between the expression of *HIF-1α*, *MDR1 *and *MRP1 *in spinal chordoma as well as their prognostic and predictive value.

## Materials and methods

### Tumors

A total of 50 primary conventional chordoma specimens between the year 2000 and 2008 (32 males and 18 females) were used for the study. The lesions were obtained from the Department of Pathology (Orthopedics Oncology Institute, Tangdu Hospital, P. R. China). 7 lesions were located in the cervical to lumbar spine and 43 in the sacrococcygeal region, at the age ranging from 31 to 80 years old (the mean age was 58). The diagnosis of all cases was confirmed by the co-expression of S-100 protein, Cytokeratin, EMA and Vimentin. Histological sections obtained at biopsy or surgically resected specimens were routinely stained with haematoxylin and eosin for diagnostic purpose. All the specimens were reviewed and diagnosed by two pathological experts. No patient in this study had undergone chemotherapy or radiotherapy before surgery. Nucleus pulposus tissues were resected in 15 patients diagnosed as lubar intervertebral disc protrusion as control. The following clinicopathological and immunohistochemical studies were conducted using sections from 10% formalin fixed paraffin-embedded tissues, highlighting the representative areas of the tumor. Light microscopic parameters and immunohistochemical analysis using the antibodies were performed in all 50 cases.

For RT-PCR, Western blot, 10 chordoma tissue samples and nucleus pulposus tissues were snap-frozen and stored at -80°C until use. Surgical samples were kept in RPMI 1640 cell culture medium before isolation of chordoma cells (within 2 h after removal).

### Cell culture

Human chordoma cell line CM-319 was derived from a case of sacral chordoma [13]. The cell line was maintained at 37°C under 5% CO_2 _in RPMI 1640 medium (Invitrogene, USA) supplemented with 10% FCS (Gibco, USA), penicillin (100 units/ml), streptomycin (100 μg/ml) and 1% (v/v) L-glutamine.

### Immunohistochemical study

The chordoma tissue samples and CM-319 cells were investigated immunohistochemically for the expression of MDR1 (monoclonal, dilution 1:500; Santa Cruz Biotechnology, USA), MRP1 (monoclonal, dilution 1:500; Santa Cruz Biotechnology, USA), HIF-1α (monoclonal, dilution 1:500; Santa Cruz Biotechnology, USA). The sections (4 μm) were deparaffinized in xylene and then rehydrated through graded alcohols to water. Antigen retrieval for all the studied sections was performed in a one-step procedure with the EDTA (PH 8.0) in a microwave oven by heating for 5 minutes. Endogenous peroxidase activity was blocked using 30% H_2_O_2 _for 30 min. Nonspecific binding was blocked with 5% goat serum in phosphate buffer solution (PBS). Sections were incubated with the primary antibodies at the reference working concentration overnight at 4°C. After washed three times with PBS, secondary antibodies, biotinylated anti-mouse or rabbit immunoglobulins (dilution 1: 50, Dako, Copenhagen, Denmark) were applied for 30 minutes at room temperature. Detection was performed using the ChemMate™ Envision +HRP/DAB kit (Dako, Copenhagen, Denmark). 3'-3'-Diaminobenzidine substrate was used as a chromogen, according to the manufacturer's instructions. Sections were counterstained with hematoxylin. Staining was evaluated independently by two pathologists.

The degree of staining was graded semi-quantitatively according to the percentage of stained cells and their staining intensity. In spinal chordoma, expression of HIF-1α, MDR1 and MRP1 was scored as follows: 0, none; 1, <10%; 2, 10-50%; and 3, >50% [14-18].

### RNA isolation and reverse transcription-polymerase chain reaction (RT-PCR)

Total RNA was isolated either from frozen tissue or CM-319 cells with Trizol reagent (Invitrogen, USA). cDNA was prepared according to standard methods: RNA was reverse-transcribed with oligo(dT) primer using 1 μg total RNA in a total volume of 20 μl containing transcription buffer, RNase Inhibitor, Prime Script™ RTase. For PCR, 30 cycles of denaturation (94°C for 45s), annealing (60°C for 45s), and elongation (72°C for 1 min) was performed using the following primer pairs for *HIF-1α *[19]: forward: 5'-TGGACTCTGATCATCTGACC-3', reverse: 5'-CTCAAGTTGCTGGTCATCAG-3', which yielded a 434-bp product. 30 cycles of denaturation (95°C for 1 min), annealing (55°C for 60s), and elongation (72°C for 1 min) were performed using the following primer pairs for *MDR1 *[20]: forward: 5'-GAATCTGGAGGAAGACATGACC-3', reverse:5'-TCCAATTTTGTCACCAATTCC-3', which yielded a 259-bp product.35 cycles of denaturation (95°C for 30s), annealing (50°C for 1 min), and elongation (72°C for 1 min) were performed using the following primer pairs for *MRP1*[21]: forward: 5'-TCAGCCCTTCCTGACAAGCT-3', reverse: 5'-TCTCTGCTGCAGGAGGTCCG-3', which yielded a 318-bp product. The *GAPDH *[22] control PCR was performed using the following primer pairs: forward: 5'-ACCACCATGGAGAAGGCTGG-3', reverse: 5'-CTCAGTGTAGCCCAGGATGC-3', which yielded a 527-bp product. For negative controls, the PCR reaction was performed without prior reverse transcription. Amplified cDNA was visualized by ethidium bromide staining on 1.5% agarose gels on a Bio-Rad gel scanner (Bio-Rad, USA).

### Western Blot

The chordoma cell line CM-319 and frozen nucleus pulposus tissues were harvested and lysed with a cold RIPA protein lysis buffer for 30 minutes on ice. The lysates were transferred to Eppendorf tubes and clarified by centrifugation at 12,000 g for 10 minutes at 4°C. The supernatant was kept in -80°C for future use. The BCA method was performed to determine the protein concentration in the supernatant. Samples (30 μg of total protein each) were boiled at 95°C for five minutes and loaded onto SDS-PAGE (5% stacking gel and 8% separating gel), followed with a separation at 80 volts for about two hours and subsequent transferred onto a nitrocellulose membrane. The membrane was blocked in 5% defatted milk for 1 hour at room temperature, and was then incubated in the primary antibodies diluted in 5% defatted milk/TBST overnight at 4°C (MDR1 1:200, mouse anti-human, Santa Cruz; MRP1, 1:200, rabbit anti-human, Santa Cruz; HIF-1α, 1:200, rabbit anti-human, Santa Cruz). The membrane was washed three times with TBST and incubated with the second antibodies for an hour at room temperature, then washed three times with TBST again. The enhanced chemiluminescene (ECL) system (Piece) was used for detection of MDR1, HIF-1α and MRP1. Protein bands were visualized and quantified using Quantity-One software (Bio-Rad USA). The MDR1, HIF-1α and MRP1 bands were visualized at an apparent molecular weight of 170, 120 and 190 kDa, respectively.

### Statistical analysis

Relationship between the expression of HIF-1α, MDR1 and MRP1 were defined using Kruskal-Wallis test (*x^2 ^*or Fisher's exact test). Correlations among three markers were described using the Spearman rank correlation test. Correlations between the expression of three markers and patient age, MIB-1 labelling index were estimated using the Mann-Whitney U test. All calculations and analyses were performed with SPSS 12.0 for Windows. Significance was considered to be *P *< 0.05.

## Results

### Expression of HIF-1α, MRP1 and MDR1 in human chordomas

**Figure 1:**
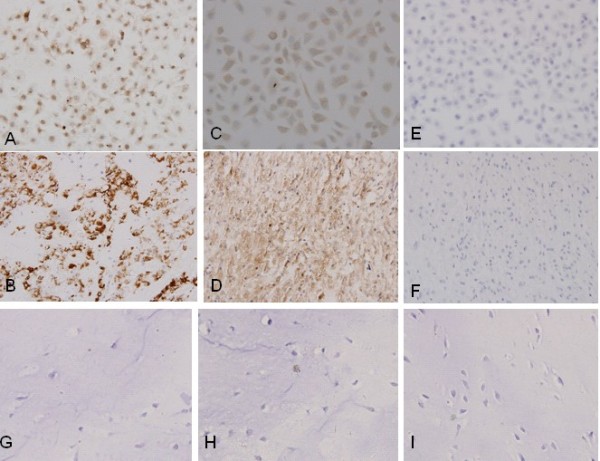
**Immunohistochemical staining of HIF-1α, MDR1 and MRP1 in chordoma, CM-319 and nucleus pulpous**. With immunohistochemical staining, the expression of chemotherapy resistant proteins using primary antibody to HIF-1α (A, B, G), MDR1 (E, F, I) and MRP1 (C, D, H) was determined in chordoma (B, D, F) and CM-319 (A, C, E). Intense membrane and cytoplasmic staining of MRP1 (×400) and cytoplasmic and nuclus staining of HIF (×400). Negative immunostaining of MDR1 was found in chordoma and CM-319. In control, negative immunostaining of HIF-1, MRP1 and MDR1 (G, H, I) was found in nucleus pulposus.

Different pattern of immunoreactivity was found as membranous or cytoplasmic staining for MDR1 and MRP1, while cytoplasmic, part of nuclear positive for HIF-1α. MDR1 positive staining was found in five (10%) of the 50 lesions which scored 1 (Figure 1E, F), and scored 0 in the remaining lesions. Thirteen of the 50 lesions were assigned to MRP1 score 0; three of the lesions scored 1; eighteen lesions scored 2; and sixteen lesions scored 3. Ten of the 50 lesions were assigned to HIF-1α score 0; four of the lesions scored 1; fourteen lesions scored 2; and twenty-two lesions scored 3. As a consequence, 37 (74%) lesions expressed MRP1 with score ≥1; 16 (32%) lesions showed strong expression with score 3 (Figure 1C, D). 40 (80%) lesions expressed HIF-1α with score ≥1; 22 (44%) lesions showed strong expression with score 3 (Figure 1A, B). Expression of HIF-1α in chordoma was much higher than that in nucleus pulposus; expressiong of MRP1 in chordoma was also much higher than that in nucleus pulposus; but expression of MDR1 in chordoma was not different from that in nucleus pulposus. (Table 1)

**Table 1:** Expression of HIF-1α, MRP1 and MDR1 in chordoma tissue and nucleus pulposus tissue

	positive	negative	positive rate	***χ***^**2**^	*P*
HIF-1α(n)					
chordoma	40	10	80%	18.55	<0.005
nucleus pulposus	3	12	20%		
MRP1 (n)					
chordoma	37	13	74%	11.10	<0.005
nucleus pulposus	4	11	26.7%		
MDR1 (n)					
chordoma	5	45	10%	0.343	>0.5
nucleus pulposus	3	12	20%		

### Correlation of antibody expression in chordomas tumors

Using Kruskal-Wallis test, we examined the relationship among MDR1, MRP1 and HIF-1α. For spinal chordoma tumors, whether primary or recurrent, we found that the overall immunoreactivity score of MRP1 or HIF-1α was higher in cases showing expression of MDR1. There was no correlation between the expression of MDR1, MRP1, HIF-1α expression and patient age, gender. There was no relationship between MDR1 expression and either MRP1 or HIF-1α expression. There was a significant correlation between HIF-1α expression and MRP1 expression level. Chordomas that had high MRP1 expression were also likely to have high HIF-1α expression. (Table 2)

**Table 2:** Correlation with the expression of HIF-1α, MRP1

		HIF-1α(n)	MRP1(n)	*r*	*P*
negative	0	10	13	0.8	<0.01
	1	4	3		
positive	2	14	18		
	3	22	16		

### RT-PCR analysis of *HIF-1α*, *MDR1* and *MRP1 *in chordoma cells

**Figure 2:**
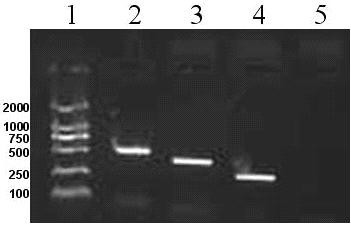
**RT-PCR analysis of *MDR1*, *HIF-1α *and *MRP1 *messenger RNA (mRNA) expression in CM-319 cell line and chordoma**. A significant *HIF-1α *and *MRP1 *mRNA expression was observed, but a negative *MDR1 *expression was observed in CM-319 cell line and chordomas. But negative expression of *MDR1*, *HIF-1α *and *MRP1 *messenger RNA (mRNA) in nucleus pulposus. Amplification of a 547-bp fragment of *GAPDH *was used as an internal control for the integrity of the isolated mRNA. Lane 1: Marker; Lane 2: GAPDH; Lane 3: HIF-1α; Lane 4: MRP1; Lane 5: MDR1.

Anaylsis of *HIF-1α*, *MDR1 *and *MRP1 *mRNA was conducted in CM-319 and chordoma by RT-PCR analysis using three pairs of primers designed for the human *HIF-1α*, *MDR1 *and *MRP1 *sequences. A 437-, 257-, 328-bp fragment should be obtained for *HIF-1α*, *MDR1 *and *MRP1 *as expected, respectively. Amplification of 547-bp fragment of *GAPDH *was used as an internal control for the integrity of the isolated mRNA. A positive *HIF-1α*and *MRP1*, but a negative *MDR1 *was observed in CM-319 cells (Figure 2).

### Western blot of HIF-1α, MDR1 and MRP1 in chordoma cells

**Figure 3:**
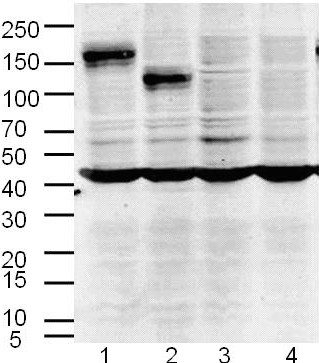
**Western blot analysis of HIF-1α, MDR1 and MRP1 protein in tumor tissues and CM-319 cell line**. Lane1: MRP1; lane2: HIF-1α; lane 3: MDR1; lane4: conditioned medium. Molecular weight markers are identificated in the left side (kD).

Expression of HIF-1α, MDR1 and MRP1 in CM-319 cells was detected by immunoblotting. The results showed no positive band with a molecular weight of 170 KD in CM-319, which indicated the negative expression of MDR1 in CM-319, but strong positive expression of HIF-1α and MRP1 at 120 KD and 190 KD in the membrane in CM-319 cells. These results were reproduced in repeat experiments of independent membrane preparations and a representative blot is shown in Figure 3.

## Discussion

Chordoma was not reported to be sensitive to chemotherapy, similar to many other low-grade malignancies. Accordingly, chemotherapy response had been reported in patients with high-grade dedifferentiated chordoma, which represented <5% of all chordoma [23].

The modern multi-modality therapeutic approach to chordoma, combining surgery with radiotherapy and chemotherapy, resulted in high cure rates even in advanced stage disease, with the pivotal role attributed to chemotherapy. However, there were still cases which exhibited either primary or secondary drug resistance with dismal outcomes [24]. Drug resistance was a major obstacle for clinical management and was attributable to several processes taking place in many kinds of tumor cells. One of these processes was the decreased accumulation of drugs within cancer cells due to drug efflux mediated by ABC multidrug transporters. Over-expression of these transporters was an adverse prognostic factor in a number of cancers. The significance of the expression of these ABC proteins in chordoma had not yet been reported.

Cellular adaptation to hypoxia was a critical step in tumor progression [25]. Hypoxia occurred during several pathophysiological processes including tumorigenesis, which was a reduction in the normal level of tissue oxygen tension. Hypoxic cancer cells might undergo a series of genetic and metabolic changes that allowed them not only to survive and proliferate but also to become more resistance to conventional therapies including ionizing radiation and chemical agents. These hypoxic adaptations made the tumors more difficult to treat and confer increased resistance to death from chemotherapy and radiotherapy. In response to hypoxia, cells altered the expression of genes that encoded protein products involved in increasing oxygen delivery and activated alternate metabolic pathways that did not require oxygen. This hypoxic response was chiefly regulated by *HIF-1α*.

Magnon's [10] findings supported a crucial role for angiogenesis inhibitors in shifting the fate of radiation-induced *HIF-1α *activity from hypoxia-induced tumor radioresistance to hypoxia-induced tumor apoptosis. Sullivan [12] determined the effects of hypoxia on multiple forms of drug-induced death in human MDA-MB-231 breast carcinoma cells. These results supported a requirement for *HIF-1 *in the adaptations leading to drug resistance and revealed that decreased drug-induced senescence was also an important contributor to the development of hypoxia-induced resistance. Nardinocchi [26] reported that the mechanistic explanation of hypoxia-induced chemoresistance involved upregulation of *HIF-1 *pathway and inhibition of the *p53 *pathway that were partly interconnected by the hypoxia-induced *HIPK2 *deregulation. They showed for the first time that hypoxia-induced *HIPK2 *deregulation was counteracted by zinc that restored *HIPK2 *suppression of *HIF-1 *pathway and reactivated *p53 *apoptotic response to drug, underscoring the potential use of zinc supplementation in combination with chemotherapy to address hypoxia and improve tumor treatment. It has been recently reported [27,28] that the transcription of *MDR1 *gene was controlled by hypoxia; *HIF-1 *binding to a putative binding site of human *MDR1 *promoter was critical for the transcription. Song [29] demonstrated that hypoxia-induced chemoresistance to cisplatin and doxorubicin in NSCLC cells was through the *HIF *pathway. *MDR1 *regulation may not be involved in hypoxia-induced chemoresistance. Combining delivery of *HIF-1α *RNAi lentiviral vector with cisplatin-related chemotherapy regimens could enable us to develop more effective strategy for NSCLC therapy. Ding [15] suggested that hypoxia induce the expression of HIF-1α and P-gp in colon carcinoma and HIF-1α expression may be associated with P-gp and interactively involved in the occurrence of tumor multidrug resistance.

In this study, we described the expression of these three different proteins associated with multidrug resistance and radiotherapy in chordoma. All the tested markers exhibited some changes in their expression pattern in chordoma compared with normal nucleus pulpous. The most prominent reduction in expression was observed for *MDR1 *which was very weakly expressed or unexpressed in more than 50% of the chordoma samples studied. To our knowledge, this was the first study on genes associated with resistance to chemotherapy and radiotherapy in spinal chordoma. The current results showed that *MRP1*was expressed in the membranous and intracellular regions; *HIF-1α *was expressed in the cell cytoplasmic and nuclear regions, whereas *MDR1 *was not expressed in the chordoma tissues or CM-319 cell.

ABC multidrug transporters also played an important role in the establishment of important biological barriers such as the placenta, the blood-brain barrier, and the blood-testes barrier. Although the over-expression of these transporters was a common phenomenon in chemoresistant tumor cells, we found that MRP1 and HIF-1α expression was upregulated in most chordoma tissues in comparison to normal tissues. It had been proposed that upregulation of ABC multidrug transporters in cancers may play a role in tumorigenesis by enhancing exposure of tissues to carcinogenic xenobiotics. Interestingly, the expression of MDR1 was not inversely expressed in the chordoma tissues.

New data on *HIF-1 *signaling and the potential for targeted therapies, including combinations of hormonal therapies for cancer and selective investigational *HIF-1α *inhibiting small molecules would be discussed. Another mechanism by which hypoxia could increase chemoresistance was to enhance the expression of *MDR1 *gene via a *HIF-1*-dependent regulation [30,31].

## Abbreviations

*HIF-1α*: hypoxia-inducible factor alpha (α) heterodimeric; *MDR1*/P-GP: multidrug resistance gene/P-glycoprotein; *MRP1*: multidrug resistance-associated protein 1

## Competing interests

The authors declare that they have no competing interests.

## Authors' contributions

ZJ and HL conceived of the study, and participated in its design and coordination and helped to draft the manuscript. YH, XQ and XC carried out the molecular genetic studies. ZL and DF participated in its design and coordination. BM and QF participated in the conception and the design of the analysis. All authors read and approved the final manuscript.
